# Is cancer mortality increasing in France?

**DOI:** 10.1054/bjoc.2001.2141

**Published:** 2001-11

**Authors:** C Hill, P Jan, F Doyon

**Affiliations:** Institut Gustave Roussy, Villejuif, 94 805, France; U521 Institut National de la Santé et de la Recherche Médicale

**Keywords:** cancer, mortality, trends, France

## Abstract

Long-term trends in cancer mortality are reported by site. Overall, cancer mortality has been decreasing in France since 1987 in the male population and since 1968 in the female population. Improvement in treatments and diagnosis should lead to persistently declining mortality rates, unless the tobacco epidemic reverses the trend in female mortality. © 2001 Cancer Research Campaign http://www.bjcancer.com


*British Journal of Cancer* (2001) **85**(11), 1664–1666
© 2001 Cancer Research Campaign
doi: 10.1054/ bjoc.2001.2141, available online at http://www.idealibrary.com on IDEAL

http://www.bjcancer.com

Short Communication

### Is cancer mortality increasing in France?

**C Hill[1], P Jan[1] and F Doyon[1,2]**

[1]Institut Gustave Roussy, 94 805 Villejuif, France; [2]U521 Institut National de la Santé et de la Recherche Médicale

**Summary** Long-term trends in cancer mortality are reported by site. Overall, cancer mortality has been decreasing in France since 1987 in the male population and since 1968 in the female population. Improvement in treatments and diagnosis should lead to persistently declining mortality rates, unless the tobacco epidemic reverses the trend in female mortality. © 2001 Cancer Research Campaign http://www.bjcancer.com

**Keywords**: cancer; mortality; trends; France

In France, as in many other developed countries, cancer is the second most common cause of death after cardiovascular diseases overall, and it is the most common cause of death in the male population since 1989. In 1997, 87 000 male deaths (32% of all deaths) were due to cancer while cardiovascular diseases accounted for 29% of all fatalities. In the female population, 56 000 deaths (22% of all deaths) were due to cancer versus 36% caused by cardiovascular diseases (http://sc8.vesinet.inserm.fr:1080/).

National mortality statistics are the only source on which the study of long-term trends in cancer can be based as there is no national cancer registry in France. The regional registries cover 11% of a population totalling 58 millions, and do not dispose of data for years prior to 1975 (Ménégoz and Chérié-Challine, 1998). We present here a summary of trends in mortality between 1950 and 1997 for the most common cancer sites.

In France, national death statistics based on death certificates are published annually by cause, sex and age in 5-year age groups. The quality of death certification has been improving over time: 18% of all death certificates bore no mention of any specified cause in 1952 versus 6% in 1997.

Estimates of the size of the population, by sex and age are published yearly by the *Institut National de la Statistique et des Etudes Economiques* (Bilan démographique, 1998) and are also available on www.ined.fr. Mortality rates have been standardised on the standard European population (www.iarc.fr).

Between 1950 and 1997, the number of cancer deaths doubled in France, increasing from 73 000 to 143 000. A large part of this rise is due to an increase from 42 to 58 million in the size of the population, and to a greater proportion of individuals aged 70 or over (from 7% to 11%). The age-adjusted mortality rate per 100 000 increased by 13% from 175 to 198 once these effects are removed.

Overall, cancer mortality rose in the male population from 200 per 100 000 inhabitants in 1950 to a maximum of 306 in 1987, and subsequently declined to 273 per 100 000 in 1997. In the female population, cancer mortality remained stable between 1950 and 1968 at around 150 per 100 000, and decreased regularly thereafter, to 123 per 100 000 in 1997 (Figure 1).

*Received 4 July 2001*
*Revised 7 August 2001*
*Accepted 7 August 2001*

*Correspondence to:* C Hill

Figure 2 depicts the trends in the male and female populations, for the main sites of cancer. Mortality is increasing for a few sites and decreasing or stable for most sites.

### Cancer sites for which mortality is increasing, at least for one sex

*Lung* cancer mortality increased markedly in the male population until the late 1980s, and has stabilised since but is still the most common cause of cancer in males (25% of cancer deaths). This pattern of progression in risk followed by stabilisation is observed in all age groups. In the female population, lung cancer mortality has doubled during the last 35 years, with an accelerated increase during the last 10 years and in the population aged 35 to 49. Notwithstanding, lung cancer remains relatively uncommon in France in the female population (7% of cancer deaths). Tobacco is the main risk factor. Its consumption rose until 1985, attaining a maximum of 6.8 g per adult (age 15 +)

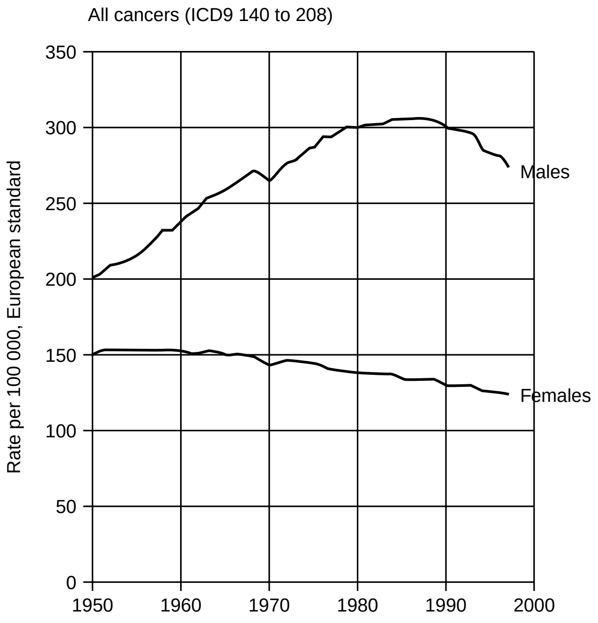

**Figure 1** Trends in cancer mortality in France

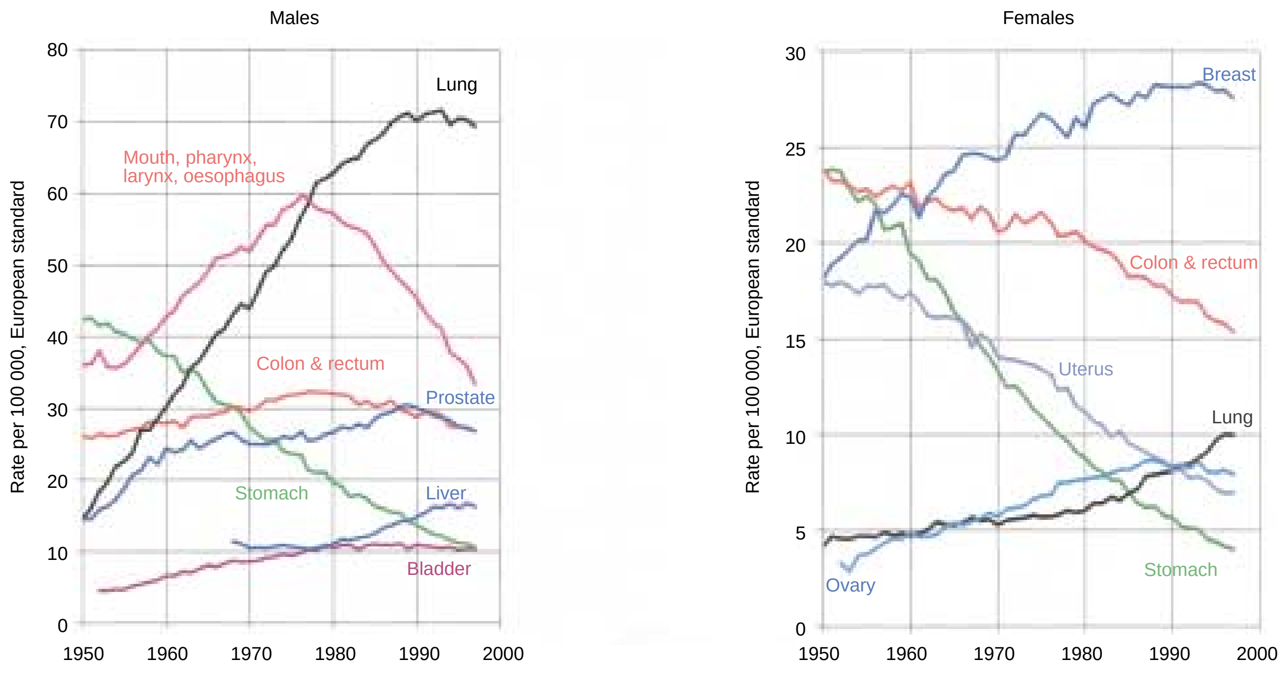

**Figure 2** Trends in cancer mortality in France for the most common sites per day, and decreased subsequently to 5.5 g in 2000. The proportion of regular smokers between 1953 and 1998 decreased from 72% to 39% in the male population and increased from 17% to 26% in the female population. The recent stability in male mortality is probably the consequence of the reduction in the proportion of smokers and of widespread use of filtered and lighter cigarettes. In 1950, less than 5% of cigarettes had a filter versus 90% today and the average tar content has markedly decreased from 35 mg in 1950 to 10 mg today, although the tar content as measured according to the International Standard does not reflect the amount inhaled by a smoker who tends to compensate for a low nicotine and tar delivery by increasing smoke intake. French women started smoking en masse in the late 1960s, which explains the recent increase recorded in tobacco consumption and low mortality due to lung cancer among women, for the time being.

Mortality also increased among less common cancers not shown on Figure 1: lymphoma, multiple myeloma, brain tumours and skin melanoma. Mortality due to *lymphoma* increased moderately until 1978 and then more markedly thereafter, with greater progression in the population aged 70 or over. Mortality due to *multiple myeloma* rose regularly until the mid 1980s and is stable since. Mortality caused by *brain tumours* increased regularly, except during the last few years where it seems to have stabilised; the increase is larger in the population aged 70 or over and from 1980 onwards, as the use of CT scan became widespread. Mortality due to *skin melanoma* has increased markedly with a concomitant decrease in mortality due to non-melanomatous skin cancer, consequently the total mortality due to skin cancer decreased until 1970 and is stable since. This suggests that the increase observed for melanoma stems essentially from better certification of the causes of death with fewer certificates mentioning ‘skin cancer not otherwise specified’.

### Cancer sites for which mortality is decreasing or stable

After a steady increase between 1950 and 1976, mortality due to *mouth*, *pharynx*, *larynx* and *oesophageal cancer* in the male population steadily returned to its 1950 level. In the female population, mortality is relatively stable for these sites. Despite the recent decrease observed in the male population, mortality due to head and neck cancer remains one of the highest in the world, among both males and females. The death rate is 9-fold higher in males than in females (ratio of standardised rates). Alcohol and tobacco are the main causes of these cancers in France (Schwartz et al, 1962; Tuyns et al, 1977; Brugère et al, 1986; Guénel et al, 1988; Launoy et al, 1997), where, traditionally, alcohol consumption is extremely high in men. In 1950, the average consumption per adult (age 15 or over) per day was 5 units (50 g of pure alcohol) compared to 1.4 units in the UK or in the USA. From 1950 to 1995, the consumption of alcohol dropped markedly to 3.2 units. This diminution explains probably a large part of the decrease in mortality, additional factors are the reduction in smoking prevalence among men and the increased consumption of fresh fruits and vegetables.

Mortality due to *stomach* cancer decreased steadily and substantially in the male and female population. This decrease has been observed in most developed countries and is attributed to a reduction in *Helicobacter pylori* infection, to an increase in consumption of fruits and vegetable (Hill, 1990) and to the generalisation of refrigerated food conservation, which has correspondingly reduced the consumption of aliments with a high salt and nitrate content.

Between 1950 and the mid 1970s, *colorectal* cancer mortality increased moderately in the male population while a slight decline was observed in the female population. Since the mid 1970s, colorectal cancer mortality has decreased and to a greater extent in the female than in the male population. This decrease may be due to a wider use of surgery as the primary treatment, screening remains marginal.

© 2001 Cancer Research Campaign

Mortality due to cancer of the uterus (*corpus plus cervix*) has decreased regularly since 1960. It is not possible to study corpus and cervix uteri cancer mortality separately before 1968 because the same code was assigned to the corpus and to 'uterus unspecified'. From 1968 onwards, cervix and corpus uteri mortality were estimated separately, by multiplying the age-specific ratio between cervix and cervix + corpus cancers by the age-specific total uterine cancer mortality ("uterus unspecified" representing 60% of the total). The decrease in mortality is greater for the cervix uteri than for the corpus uteri, and is certainly at least partly attributable to cervical cancer screening which has been largely used for more than 20 years; in 1991, 79% of the women aged 25 to 54 reported having had a smear in the previous 3 years.

*Prostate* cancer mortality has been decreasing moderately and regularly since 1989. It increased markedly in the 1950s and moderately between 1960 and 1989. This rise was observed only in the population aged 85 + which accounts for one third of the prostate cancer-related deaths; it may be explained by an improvement in the quality of the diagnosis and death certification.

*Ovary* cancer mortality has been decreasing moderately since 1987. The increase observed before 1987 is probably largely attributable to improvements in the diagnosis. The recent decrease is partly explained by an extensive use of oral contraceptives since 1976.

Since 1986, *breast* cancer mortality is stable; it had increased markedly before. These trends are observed in all age groups. Breast cancer is by far the overriding cause of cancer-related mortality in the female population (19% of cancer deaths). Its incidence was found to have increased by 60% between 1975 and 1995 (Ménégoz and Chérié-Challine, 1998). This increase in incidence is mainly due to screening which has been freely accessible in France for many years. In 1991, 27% of French women aged 40 to 69 reported having had a mammogram in the last 3 years versus 7% of the women in the UK. In 1993, 56% of women between age 50 and 69 reported having had a mammogram in the last 3 years versus 73% in 1999. The decrease in mortality observed recently in the UK and in the USA (Peto et al, 2000) is not observed in France where mortality is much lower than in the UK, may be because breast cancer has been treated more aggressively in France for a long time. Tamoxifen has been prescribed to women having a breast cancer after menopause with positive oestrogen receptors since the early 1980s and chemotherapy has been used extensively in France since 1985.

Since 1992, *liver* cancer mortality has stabilised in the male population, after a moderate increase. Liver cancer mortality decreased between 1968 and 1984 in the female population, and has stabilised since. The increase observed in the male population prior to 1992, could be due to better diagnosis of liver cancer in cirrhotic patients, and is unlikely to be related to hepatitis C infection since there has been no increase in women. The proportion of liver cancers that are not specified as primary on the death certificates has declined regularly between 1968 and 1997 from 87% to 21% for males and from 90% to 26% for females, and the trends analysed include these liver cancers unspecified as primary or secondary.

After regularly increasing, *bladder* cancer mortality has stabilised in the male and female population since 1977.

Among less common sites of cancer, it is noteworthy that renal cancer mortality has stabilised and that leukaemia and thyroid cancer mortality are decreasing. *Renal* cancer mortality rate has been stable for the last several years. *Leukaemia* mortality has been decreasing since the late 1970s after an earlier increase. *Thyroid* cancer mortality has been decreasing since the mid 1980s, after a slight increase. Its incidence increased markedly between 1975 and 1995 because of improved diagnosis leading to the discovery of small nodules. This increase cannot be attributed to the 1986 Chernobyl accident because it began 10 years before the accident and is not associated with an increase in mortality.

### CONCLUSION

Overall, cancer mortality has increased by 13% since 1950. Among males, mortality increased between 1950 and 1987, followed by a decrease. Among females, cancer mortality was stable between 1950 and 1968 and since then has decreased moderately.

The initial increase among males is largely due to tobacco. The decrease observed afterwards is attributed to reduced exposure to alcohol and other carcinogens such as *Helicobacter pylori* infection, to an increase in protective factors such as fruit and vegetable consumption, and to improvements in treatments and diagnosis. Further improvements in therapy or screening and use of new diagnostic tools should lead to persistently declining mortality rates, unless the tobacco epidemic reverses the trend in female mortality.

### ACKNOWLEDGEMENTS

We thank Professor Sir Richard Doll for helpful comments and Lorna Saint-Ange for linguistic assistance.

### REFERENCES

Bilan démographique 1998 (1999) In: Institut National de la Statistique et des Etudes Economiques. *Bulletin Mensuel de Statistique* **1**: 143–152

Brugère J, Guénel P, Leclerc A and Rodriguez J (1986) Differential effects of tobacco and alcohol in cancer of the larynx, pharynx and mouth. *Cancer* **57**: 391–395

Guénel P, Chastang JF, Luce D, Leclerc A and Brugère J (1988) A study of the interaction of alcohol drinking and tobacco smoking among French cases of laryngeal cancer. *J Epidemiol Community Health* **42**: 350–354

Hill C (1990) Overview of available data on diet and cancer mortality in France. *Tumori* **76**: 299–305

Launoy G, Milan CH, Faivre J, Pienkowski P, Milan CI and Gignoux M (1997) Alcohol, tobacco and oesophageal cancer: effects of the duration of consumption, mean intake and current and former consumption. *Br J Cancer* **75**: 1389–1396

Ménégoz F and Chérié-Challine L (1998) Le cancer en France: incidence et mortalité. Paris: La documentation française

Peto R, Boreham J, Clarke M, Davies C and Beral V (2000) UK and USA breast cancer deaths down 25% in year 2000 at ages 20–69 years. *Lancet* **355**: 1822

Schwartz D, Lellouch J, Flamant R and Denoix PF (1962) Alcool et cancer. Résultats d'une enquête rétrospective. *Rev Franc Et Clin Biol* **7**: 590–604

Tuyns AJ, Péquignot G and Jensen OM (1977) Le cancer de l'œsophage en Ille et Vilaine en fonction des niveaux des consommation d'alcool et de tabac. *Bull Cancer* **64**: 45–60

*© 2001 Cancer Research Campaign*

